# Long-Term Efficacy and Safety of Paliperidone 6-Month Formulation: An Open-Label 2-Year Extension of a 1-Year Double-Blind Study in Adult Participants With Schizophrenia

**DOI:** 10.1093/ijnp/pyad028

**Published:** 2023-07-22

**Authors:** Dean Najarian, Ibrahim Turkoz, R Karl Knight, Silvana Galderisi, Hector F Lamaison, Piotr Zalitacz, Suresh Aravind, Ute Richarz

**Affiliations:** Janssen Scientific Affairs, LLC, Titusville, New Jersey, USA; Janssen Research & Development, LLC, Titusville, New Jersey, USA; Janssen Research & Development, LLC, Titusville, New Jersey, USA; University of Campania “Luigi Vanvitelli,” Naples, Italy; Department of Psychiatry, National University of La Plata (UNLP), Buenos Aires, Argentina; Head of Psychiatric Unit, Gorlice Specialist Hospital, Gorlice, Poland; Janssen Research & Development, LLC, Titusville, New Jersey, USA; Dural Consulting LLC, St Petersburg, Florida, USA (Dr Aravind); Janssen Research & Development, Cilag Int., Gubelstrasse, Zug, Switzerland

**Keywords:** Long-term efficacy, schizophrenia, long-acting injectable, open-label extension, paliperidone palmitate 6-month

## Abstract

**Background:**

Paliperidone palmitate 6-month (PP6M) demonstrated noninferiority to paliperidone palmitate 3-month in preventing relapse in patients with schizophrenia in a phase 3 double-blind (DB) study (NCT03345342). Here, we report long-term efficacy and safety results from a 2-year single-arm, open-label extension (OLE; NCT04072575) of this DB study.

**Methods:**

Participants who completed the DB study without relapse were enrolled and followed-up every 3 months up to 2 years. Participants received 4 PP6M gluteal injections (700/1000 mg eq.) at baseline, 6-month, 12-month, and 18-month visits. Efficacy endpoints included assessment of relapse, Positive and Negative Syndrome Scale total score, Personal and Social Performance score, and Clinical Global Impression-Severity scale change from baseline. Safety was assessed by treatment-emergent adverse events (TEAEs), physical examinations, and laboratory tests.

**Results:**

Of 178 participants enrolled, 154 (86.5%) completed the OLE (mean age: 40.4 years, men: 70.8%; mean duration of PP6M exposure during OLE: 682.1 days). Overall, 7/178 (3.9%) participants relapsed between 20 and 703 days after enrolment. Mean (SD) changes from baseline to endpoint were as follows: Positive and Negative Syndrome Scale total score, 0.7 (8.22); Clinical Global Impression-Severity, 0.0 (0.51); and Personal and Social Performance Scale, 0.5 (7.47). Overall, 111/178 participants (62.4%) reported ≥1 TEAE; most common (>5%) TEAEs were headache (13.5%) and increased blood prolactin/hyperprolactinemia (18.0%); 8/178 (4.5%) participants experienced serious TEAEs, and 6/178 (3.4%) participants withdrew due to TEAEs. No deaths were reported.

**Conclusions:**

The relapse rate observed with PP6M during the 2-year OLE was low (3.9%). Clinical and functional improvements demonstrated in the DB study were maintained during OLE, and no new safety concerns were identified.

**Trial registration:**

ClinicalTrials.gov Identifier: NCT04072575; EudraCT number: 2018-004532-30.

**Significance Statement**

Long-acting injectable (LAI) antipsychotic medications are an important treatment option for patients with schizophrenia, particularly those at risk of non-adherence to daily oral antipsychotic medications or challenges for frequent health care. The administration frequency of LAIs ranges from every 2 weeks to every 3 months, except for paliperidone palmitate 6-month formulation (PP6M), which has the longest available dosing-option of twice-yearly administration. PP6M is approved for treatment of schizophrenia in adults after adequate treatment with its 1-month (PP1M) or 3-month (PP3M) counterparts. The phase 3 double-blind study of PP6M (NCT03345342) demonstrated noninferiority of PP6M to PP3M in prevention of relapses, with no new safety concerns. The current study is a 2-year open-label extension of PP6M double-blind study and demonstrated long-term efficacy and safety with low patient relapse rates. Thus PP6M, with only 2 dose administrations annually, is a potential option for long-term treatment of patients with schizophrenia, especially if adherence or healthcare access are concerns.

## INTRODUCTION

Poor treatment adherence associated with frequent relapses along with functional deterioration is a characteristic feature in the clinical course of schizophrenia (Morken et al., 2008; Higashi et al., 2013; Anderson et al., 2017). With most patients requiring long-term treatment with antipsychotic medications, long-acting injectable (LAI) formulations provide an advantage over oral antipsychotics with improved treatment outcomes, including improved medication adherence, lower rates of rehospitalizations, improved relapse rates and functional outcomes, and decreased health-care costs (Brissos et al., 2014; Marcus et al., 2015; Correll et al., 2016; Moreno et al., 2020).

Paliperidone palmitate, an atypical/second-generation antipsychotic, is a LAI formulation of paliperidone that is currently available as 1-month (PP1M), 3-month (PP3M), and 6-month (PP6M) injectable formulations. Both PP1M and PP3M have previously demonstrated efficacy in symptom control, relapse prevention, and reduction in hospitalizations in patients with schizophrenia (Hough et al., 2010; Gopal et al., 2011; Berwaerts et al., 2015; Savitz et al., 2016). The recently developed PP6M formulation is the first LAI with a substantially longer dosing interval of 6 months, enabling just 2 injections annually for patients who have been adequately treated on PP1M or PP3M (Najarian et al., 2022).

A recent global phase 3, randomized, double-blind (DB), active-controlled, relapse prevention study (hereafter referred to as DB study) demonstrated noninferiority of PP6M (700 and 1000 mg eq. doses) to PP3M (350 and 525 mg eq. doses) in preventing relapse in participants with schizophrenia previously stabilized on PP1M or PP3M. The DB study suggested a comparable efficacy of PP6M with its 3-month counterpart (PP3M) for participants who remained relapse free at the end of the 12-month DB treatment. Safety findings for PP6M were consistent with the known profile of paliperidone palmitate in this study, and no new concerns specific for PP6M were identified (Najarian et al., 2022). The pharmacokinetic characteristics of PP1M, PP3M, and PP6M and their dose equivalencies have been published elsewhere (Cleton et al., 2014; Ravenstijn et al., 2016; Najarian et al., 2022).

The current study is a 2-year, single-arm, open-label extension (NCT03345342; hereafter referred to as open-label extension [OLE] study) of the DB study and was aimed to evaluate the long-term efficacy and safety of PP6M in participants who remained relapse free in the DB study.

## METHODS

### Study Design and Participants

**Figure 1.**
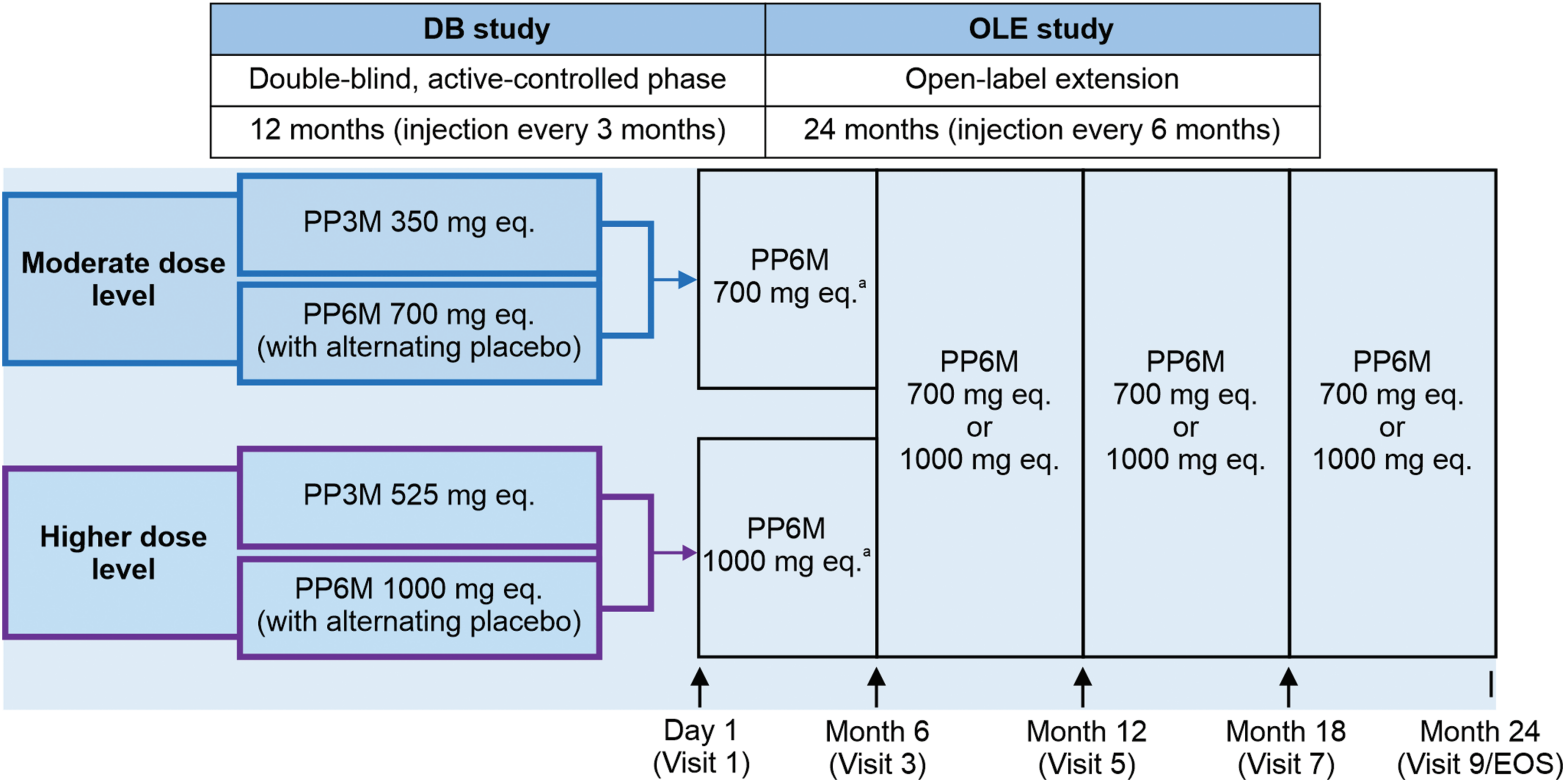
Study design of the double-blind study and open-label extension study. ^a^The initial dose of PP6M in OLE study was determined based on the dose level (“moderate” or “higher”) that the participant was receiving during the DB phase. The PP6M dose level was to be adjusted (to 700 or 1000 mg eq.) later in the study (at visits 3, 5, and 7), based on clinical judgment. However, the long-acting nature of PP6M means a dose change could take many months to become apparent. ↑, PP6M injection; DB, double-blind; EOS, End of Study; OLE, open-label extension; PP3M, paliperidone palmitate 3-month formulation; PP6M, paliperidone palmitate 6-month formulation.

This single-arm OLE study was conducted between September 2019 and May 2022 in 6 (Argentina, Hong Kong, Italy, Poland, Russia, and Ukraine) of the 20 countries that had previously participated in the DB noninferiority study. Eligible participants (aged 18–70 years with a diagnosis of schizophrenia per Diagnostic and Statistical Manual of Mental Disorders, 5th edition for ≥6 months before screening and a Positive and Negative Syndrome Scale [PANSS] total score <70 points at screening; Najarian et al., 2022) who completed the DB study on PP3M or PP6M without a relapse and wished to continue treatment with PP6M were enrolled (**Figure 1**). Enrollment into the OLE study was optional and at the participant’s or investigator’s discretion. Participants received a total of 4 injections of 700 mg eq. (3.5 mL) or 1000 mg eq. (5.0 mL) doses of PP6M (supplied in prefilled syringes) administered at baseline, 6-month, 12-month, and 18-month visits (**Figure 1**). All PP6M injections were to be administered in the gluteal muscle using the dorsogluteal route (upper outer quadrant) and alternately on either side (left or right) of the body. The initial dose of PP6M received during the OLE (i.e., at visit 1) was determined based on the participant’s dose level (moderate or high) during the DB study. Since treatment assignment (i.e., to PP6M vs PP3M) during the DB study was blinded when visit 1 occurred, dosing was fixed for all participants at this visit to ensure that those participants treated with PP3M during the DB phase would initiate treatment with PP6M at an equivalent dose. Flexible dosing was permitted at subsequent visits; however, given the slow rate of change in paliperidone blood levels expected over time, it may take weeks or months for the desired effect of a dose change to occur. Participants were followed for up to 2 years in the OLE phase with visits every 3 months, consistent with real-world practice.

For additional analysis of safety and efficacy, OLE participants were categorized into 2 subgroups, PP3M/PP6M and PP6M/PP6M, indicating participants who had received PP3M and those who received PP6M in the DB study before the OLE.

The concomitant use of anti-extrapyramidal symptom medications, benzodiazepines, sleep aids, and oral antipsychotics were allowed during the OLE. The duration and dose of antipsychotic supplementation was dependent on symptom exacerbation and per the investigator’s judgment (supplementary **Table 1**).

### Study Assessments

Efficacy endpoints included assessment of relapse events and change from baseline to the endpoint (Month 24) in PANSS (Kay et al., 1987) total score, Personal and Social Performance (PSP) (Morosini et al., 2000) score, and Clinical Global Impression-Severity (CGI-S) (Busner and Targum, 2007) score. A relapse was defined as presence of 1 or more episodes of psychiatric hospitalization, emergency department treatment due to schizophrenia symptoms, participant behavior resulting in harm (self-injury, suicide, harm to another person, or property damage), or suicidal or homicidal ideation. Secondary endpoints included change from baseline to endpoint in PANSS subscale scores and symptomatic remission scores.

Safety was assessed by incidence of treatment-emergent adverse events (TEAEs; coded using Medical Dictionary for Regulatory Activities version 22.1), serious TEAEs, mental status examination, clinical laboratory tests, vital sign measurements, physical examinations, and evaluation of the injection site. The Columbia Suicide Severity Rating Scale was performed if suicidal ideation was identified during the mental status examination. Extrapyramidal symptom (EPS) rating scales and 12 lead electrocardiogram assessments were conducted at the investigator’s discretion. All clinical laboratory tests, including prolactin levels, were performed on fasted samples and were analyzed by a local laboratory.

### Statistical Analysis

No formal sample size determination was performed for this study. The sample size was determined by the number of participants who completed the DB study without relapse and were willing to participate in this OLE.

Efficacy and safety analyses were conducted on an intent-to-treat analysis set, which included all participants who received ≥1 dose of the study medication. Descriptive statistics (mean, SD, median, range [minimum and maximum]) were provided for the CGI-S, PSP, and PANSS over time, including all assessment time points from the baseline of the DB phase to the end of study or early withdrawal visit of this OLE study.

Relapse rates and number and percentage of participants achieving symptomatic remission were summarized at month 12, month 24, and at the end of study or early withdrawal visit. For single observations, transitory symptomatic remission was defined as having a simultaneous score of mild or less (≤3 points) on the following 8 items from the PANSS: the positive-symptom items P1 (delusions), P2 (conceptual disorganization), P3 (hallucinatory behavior); the negative symptom items N1 (blunted affect), N4 (social withdrawal), and N6 (lack of spontaneity); and the general-psychopathology items G5 (mannerisms/posturing) and G9 (unusual thought content). TEAEs were summarized descriptively.

### Ethics

This clinical study was conducted in accordance with the principles of the Declaration of Helsinki and local laws and regulations. Written informed consent was provided by all participants before any study procedures took place. The study protocol and all amendments were reviewed by the independent ethics committee and/or institutional review board for each study center.

## RESULTS

### Participant Disposition and Baseline Characteristics

A total 618 of 702 participants completed the DB study and 571 participants completed the 12-month DB phase without a relapse. In the centers where the open label study was conducted, 178 participants without relapse elected to continue the treatment and enter the OLE study. Of these participants, 154 (86.5%) completed the 2-year OLE study (**Table 1**). The median age was 39 years (range: 19–69 years), with most participants (75.8%) aged between 26 and 50 years. Most participants were men (70.8%) and White (98.9%). The PP3M/PP6M subgroup included 57 participants, and the PP6M/PP6M subgroup had 121 participants. The demographic characteristics were similar between the subgroups (**Table 1**).

**Table 1.** Participant Demographics and Baseline Characteristics

Characteristic	PP3M/PP6M (n = 57)	PP6M/PP6M (n = 121)	Total (N = 178)
Age,^a^ mean (SD), y	41.3 (9.74)	40.0 (11.23)	40.4 (10.76)
Sex, n (%)			
Men	43 (75.4)	83 (68.6)	126 (70.8)
Race, n (%)			
White	56 (98.2)	120 (99.2)	176 (98.9)
Asian^b^	1 (1.8)	1 (0.8)	2 (1.1)
Ethnicity			
Hispanic or Latino, n (%)	13 (22.8)	35 (28.9)	48 (27.0)
Not Hispanic or Latino	44 (77.2)	86 (71.1)	130 (73.0)
Baseline weight, mean (SD), kg	81.7 (15.96)	85.3 (15.69)	84.2 (15.82)
Baseline BMI, mean (SD), kg/m^2^	27.7 (4.93)	28.7 (4.89)	28.4 (4.91)
Baseline BMI category, kg/m^2^, n (%)			
Normal (<25)	16 (28.1)	25 (20.7)	41 (23.0)
Overweight (25 to <30)	20 (35.1)	43 (35.3)	63 (35.4)
Obese (≥30)	21 (36.8)	53 (43.8)	74 (41.6)
Age at schizophrenia diagnosis, mean (SD), y	29.3 (8.73)	27.5 (9.21)	28.1 (9.07)
Prior hospitalization,^c^ n (%)			
N	43	89	132
None	21 (48.8)	42 (47.2)	63 (47.7)
Once	12 (27.9)	28 (31.5)	40 (30.3)
Twice	7 (16.3)	13 (14.6)	20 (15.2)
Three times	3 (7.0)	4 (4.5)	7 (5.3)
Four times or more	0	0	0
PANSS, mean (SD)			
Baseline	50.2 (10.77)	49.4 (10.40)	49.6 (10.50)
Baseline (DB)	53.1 (10.05)	53.4 (9.72)	53.3 (9.80)
PSP, mean (SD)			
Baseline	71.5 (11.84)	71.5 (10.79)	71.5 (11.10)
Baseline (DB)	69.8 (11.69)	68.7 (12.10)	69.0 (11.95)
CGI-S, mean (SD)			
Baseline	2.9 (0.83)	2.8 (0.78)	2.8 (0.80)
Baseline (DB)	2.9 (0.84)	3.0 (0.77)	3.0 (0.80)

Abbreviations: BMI, body mass index; CGI-S, Clinical Global Impression-Severity; DB, double-blind; PANSS, Positive and Negative Symptom Scale; PP3M, paliperidone palmitate 3-month formulation; PP6M, paliperidone palmitate 6-month formulation; PSP, Personal and Social Performance.

^a^Age at screening.

^b^Asian subcategories include Chinese, Korean, Japanese, Filipino, Asian Indian, Thai, Malaysian, and Asian (other).

^c^Number of hospitalizations for psychosis within 24 months before study start.

The mean (SD) duration of exposure was 682.1 (150.8) days, and the mean (SD) dose was 875.7 (140.15) mg eq. Most participants (158 participants [88.8%]) received all 4 doses of PP6M. Overall, 24 (13.5%) participants withdrew before study completion, with withdrawal by participant (7.9%) and adverse events (3.9%) being the common reasons for discontinuations.

### Efficacy

**Figure 2.**
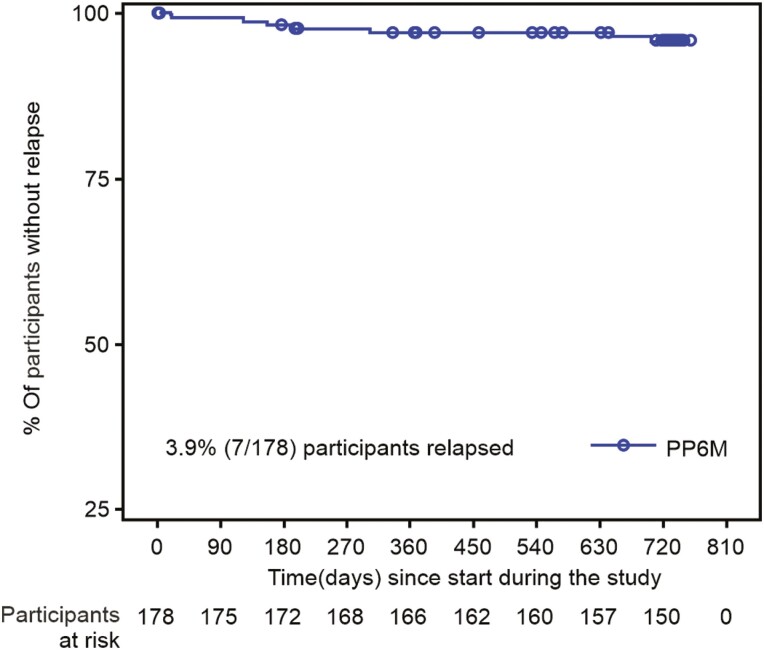
Kaplan Meier estimates of time to relapse during the open-label extension. PP6M, paliperidone palmitate 6-month formulation.

Overall, 7/178 (3.93%) participants relapsed during the study between days 20 and 703 of study enrolment. Time to relapse is depicted in a Kaplan Meier plot (**Figure 2**).

**Figure 3.**
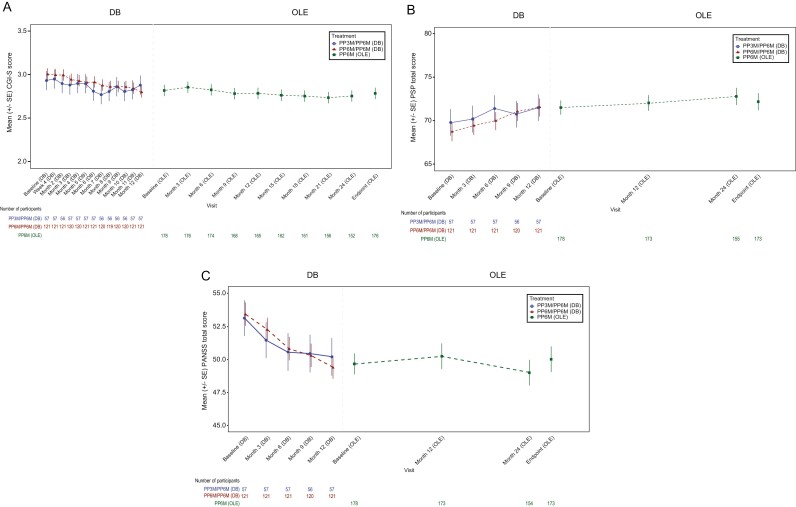
Mean (±SE) (A) CGI-S, (B) PSP total, (C) PANSS total scores, over time from double-blind study to end of open-label extension. CGI-S, Clinical Global Impression-Severity; DB, double-blind; OL, open-label; PANSS, Positive and Negative Symptom Scale; PSP, Personal and Social Performance; PP3M, paliperidone palmitate 3-month product; PP6M, paliperidone palmitate 6-month product; SE, standard error. Note: Lower scores in PANSS and CGI indicate improvement and higher scores in PSP denote an improvement.

During this 2-year follow-up, participants maintained the clinical and functional improvement achieved during the DB study, as reported by PANSS, CGI-S, and PSP scores (**Table 2**; **Figure 3**). Mean (SD) change from baseline to endpoint for PANSS total score was 0.7 (8.22), CGI-S was 0.0 (0.51), and PSP scale was 0.5 (7.47). These efficacy results were generally consistent across the PP3M/PP6M and PP6M/PP6M subgroups (supplementary **Table 2**).

**Table 2.** Summary of Efficacy Endpoints: Change From Baseline to Endpoint of Open-label Extension (Intent-to-Treat Set)

Test	Baseline	Endpoint	Change from Baseline
CGI-S scale	2.8 (0.80)	2.8 (0.92)	0.0 (0.51)
Severity of illness, n (%)			
Normal	10 (5.6)	16 (9.1)	–
Borderline	45 (25.3)	47 (26.7)	-–
Mild	90 (50.6)	73 (41.5)	–
Moderate	33 (18.5)	38 (21.6)	–
Marked	0	2 (1.1)	–
Severe	0	0	–
Most extreme	0	0	–
PSP scale	71.5 (11.10)	72.1 (12.75)	0.5 (7.47)
PSP score shift, n			
Poor (≤30)	0	0	
Variable (>30–≤70)	78	74	
Good (>70)	95	99	
PANSS total score, mean (SD)	49.6 (10.50)	50.0 (12.84)	0.7 (8.22)
PANSS subscale scores, mean (SD)			
Positive subscale	10.3 (2.82)	10.6 (3.91)	0.3 (3.10)
Negative subscale	15.6 (4.39)	15.5 (4.84)	0.0 (2.71)
General-psychopathology subscale	23.7 (5.14)	23.9 (6.32)	0.3 (4.51)
PANSS Marder standardized factor scores, mean (SD)			
Positive symptoms	13.0 (3.98)	13.0 (4.63)	0.1 (3.16)
Negative symptoms	14.2 (3.97)	14.3 (4.26)	0.2 (2.64)
Disorganized thoughts	12.6 (3.76)	12.5 (4.16)	0.0 (2.67)
Uncontrolled hostility/excitement	4.7 (1.15)	4.8 (1.90)	0.2 (1.62)
Anxiety/depression	5.2 (1.63)	5.4 (1.89)	0.2 (1.90)

Abbreviations: CGI-S, Clinical Global Impression-Severity; DB, double-blind; PANSS, Positive and Negative Symptom Scale; PSP, Personal and Social Performance; SD, standard deviation.

Symptomatic remission, as measured by PANSS subscale scores, occurred in 81.5% of all participants at 12 months and 84.4% at 24 months, with the PP6M/PP6M group showing slightly higher remission rates at all timepoints (**Table 3**).

**Table 3.** PANSS Symptomatic Remission During Open-Label Extension

Remission Status^a^ n (%)	PP3M/PP6M (n = 57)	PP6M/PP6M (n = 121)	Total (N = 178)
Month 12			
No	13 (23.2)	19 (16.2)	32 (18.5)
Yes	43 (76.8)	98 (83.8)	141(81.5)
Month 24			
No	10 (18.9)	14 (13.9)	24 (15.6)
Yes	43 (81.1)	87 (86.1)	130 (84.4)
Endpoint			
No	10 (17.9)	18 (15.4)	28 (16.2)
Yes	46 (82.1)	99 (84.6)	145 (83.8)

Abbreviations: PANSS, Positive and Negative Symptom Scale; PP3M, paliperidone palmitate 3-month formulation; PP6M, paliperidone palmitate 6-month formulation.

^a^Remission is defined as having a score ≤3 on all these PANSS items: P1, P2, P3, N1, N4, N6, G5, and G9.

### Safety

Overall, 111/178 participants (62.4%) experienced ≥1 TEAE (**Table 4**). The most commonly reported TEAEs (>5%) included headache (13.5%) and increased blood prolactin/hyperprolatinemia (18.0%). Incidences of headache were similar across subgroups, while the incidence of increased blood prolactin/hyperprolactinemia was slightly higher (19.0%) in the PP6M/PP6M subgroup compared with the PP3M/PP6M subgroup (15.8%).

**Table 4.** Treatment-Emergent Adverse Events in ≥5% of Participants in Any Group in the Open-Label Extension (Intent-to-Treat Set)

n (%)	PP3M/PP6M (n = 57)	PP6M/PP6M (n = 121)	Total (N = 178)
≥1 TEAEs	35 (61.4)	76 (62.8)	111 (62.4)
Headache	8 (14.0)	16 (13.2)	24 (13.5)
Blood prolactin increased^a^	5 (8.8)	14 (11.6)	19 (10.7)
Hyperprolactinemia^a^	4 (7.0)	9 (7.4)	13 (7.3)
Diarrhea	4 (7.0)	7 (5.8)	11 (6.2)
Weight increased	4 (7.0)	5 (4.1)	9 (5.1)
Nasopharyngitis	2 (3.5)	7 (5.8)	9 (5.1)
Blood creatinine phosphokinase increased	3 (5.3)	2 (1.7)	5 (2.8)
Insomnia	3 (5.3)	2 (1.7)	5 (2.8)

Abbreviations: PP3M, paliperidone palmitate 3-month formulation; PP6M, paliperidone palmitate 6-month formulation; TEAE, treatment-emergent adverse event.

^a^It was investigators’ discretion in how they categorized “blood prolactin increased” and “hyperprolactinaemia” as TEAE.

A total of 8/178 participants (4.5%) experienced serious TEAEs (**Table 5**). The most frequently reported serious TEAE was worsening of schizophrenia in 4 participants (2.2%). Overall, 6/178 (3.4%) participants reported TEAEs leading to treatment discontinuation; the most frequent was worsening of schizophrenia (n = 4 participants: 2.2%). No deaths occurred during the study.

**Table 5.** Summary of Serious Treatment-Emergent Adverse Events in Open-Label Extension

n (%)	PP3M/PP6M n = 57	PP6M/PP6M n = 121	Total N = 178
≥1 Serious TEAEs	2 (3.5)	6 (5.0)	8 (4.5)
Schizophrenia	1 (1.8)	3 (2.5)	4 (2.2)
Psychiatric symptom	0	1 (0.8)	1 (0.6)
Hallucination, auditory	1 (1.8)	0	1 (0.6)
Colon cancer	0	1 (0.8)	1 (0.6)
Metastases to peritoneum	0	1 (0.8)	1 (0.6)
Nephrotic syndrome	0	1 (0.8)	1 (0.6)

Abbreviations: PP3M, paliperidone palmitate 3-month formulation; PP6M, paliperidone palmitate 6-month formulation; TEAE, treatment-emergent adverse event.

A low incidence of EPS-related TEAEs was noted (n = 4 participants, 2.2%; all events were new onset). Tremor was the most commonly reported EPS-related TEAE (n = 3 participants, 1.7%).

Evaluation of injection site over time, as assessed by erythema/redness, induration/swelling, and tenderness, showed that all parameters were absent or mild at baseline; erythema/redness was absent at endpoint, and induration/swelling and tenderness were mild or absent at endpoint for most participants and severe in 1 participant each (supplementary **Table 3**). Injection site-related TEAEs (≥1) were observed in 7 participants (3.9%); most common was injection site pain (n = 4 participants, 2.2%). Extremity pain was reported in 2 participants (1.1%); of these, 1 participant reported pain in the right arm (though for this participant, PP6M was injected in the left arm; deltoid injection was a protocol deviation), and the other reported right leg pain. The mean (SD) values for the participant’s evaluation of injection site pain, as measured by the Visual Analog Scale (100 mm), increased from baseline (10.1 [12.85]) to month 6 (11.3 [13.28]) and month 12 (12.6 [15.45]), and then decreased over time through month 24 (4.6 [8.63]). Only 1 participant (0.6%) reported injection site pain as TEAE.

Median prolactin levels remained relatively stable in men throughout the study, whereas in women, median prolactin levels steadily increased from baseline to month 12 and then steadily declined again between months 12 and 24 (supplementary **Figure 1**). Substantial individual fluctuations in prolactin values were observed in women, which increased the mean value.

Four participants (2.2%) reported diabetes mellitus and hyperglycemia-related TEAEs (of which 1 event is reported as new onset of diabetes); the most common was increased blood glucose (2 participants, 1.1%). There were no clinically meaningful changes in body weight, body mass index, or waist circumference.

## DISCUSSION

In this 2-year OLE study in participants with schizophrenia who had remained relapse free in the DB study and who either transitioned from PP3M to PP6M (PP3M/PP6M) or maintained the 6-month formulation (PP6M/PP6M), PP6M demonstrated long-term efficacy and safety.

The clinical improvements observed during the DB study were maintained in the OLE, as demonstrated by stable scores on PANSS, CGI-S, and PSP scales over the 2-year period with very few cases of relapses (3.9%). These efficacy results were generally consistent across the PP3M/PP6M and PP6M/PP6M subgroups. When efficacy parameters were examined from baseline to endpoint of the OLE as well as from DB study baseline to OLE endpoint, PP6M demonstrated an increased efficacy in the maintenance treatment of participants with schizophrenia (supplementary **Table 4**). Overall, efficacy results of PP6M shown in this OLE study are aligned with the previous DB study of PP6M (Najarian et al., 2022) and with the known profile of other paliperidone palmitate formulations (Hough et al., 2010; Kramer et al., 2010; Gopal et al., 2011; Berwaerts et al., 2015; Savitz et al., 2016).

The study completion rate (86.5%) observed in this 2-year OLE was comparable with those observed in the 1-year DB study (completion rate: PP6M, 87.0%; PP3M, 90.2%). However, the relapse rate (3.9%) in the OLE was lower than those observed in the DB study (relapse rate: PP6M, 7.5%; PP3M, 4.9%) (Najarian et al., 2022). The selection criteria of including participants who remained relapse free during the DB phase may have contributed to the lower relapse rates.

The follow-up frequency in the current study was every 3 months. In routine clinical practice, the frequency of patient visits should be based on clinician’s judgement and not the frequency of injection interval. In this OLE study with PP6M (administered at doses of 700 or 1000 mg eq.), no new safety signals were identified and no deaths were reported. Overall, the safety of PP6M was consistent with the known profile of paliperidone palmitate (Hough et al., 2010; Kramer et al., 2010; Gopal et al., 2011; Berwaerts et al., 2015; Savitz et al., 2016). The incidences of TEAEs were similar across the PP3M/PP6M and PP6M/PP6M subgroups.

Low rates (4.5%) of serious TEAEs were observed across all groups. The serious TEAEs reported were mainly psychiatric disorders and were indicative of worsening of the underlying disease. Incidence of EPS-related TEAEs was also low (2.2%) in this study.

As expected, prolactin levels in females were higher than males. However, there were also substantial fluctuations observed in prolactin values in females, which increased the mean value. However, none of the TEAEs related to prolactin levels were considered serious by investigators. The higher proportion of males in the study may have had a confounding effect on prolactin reporting (Bioque et al., 2020). Serum elevations did not appear to correlate with reporting of prolactin-related symptoms.

Although the volume of PP6M doses was 3.5 or 5 mL, which requires gluteal muscle administration, the incidence of injection site reaction was low (3.9%). This was confirmed by the low mean score on participant-reported Visual Analog Scale for injection site pain during the study. No clinically meaningful changes in vital signs and laboratory analytes were noted from OLE baseline to endpoint. Injection site evaluation over time showed that all the assessed parameters were either absent or mild for most participants, except for one.

The safety and efficacy data described in this study support the use of LAIs in schizophrenia treatment. To date, PP6M has the longest dosing duration of current antipsychotic agents, and it is available to participants who have demonstrated adequate treatment with the PP1M or PP3M. Evidence suggests the longer the half-life of the antipsychotic used in the treatment, the longer the patient’s time to relapse after stopping the medication. In a study comparing oral paliperidone with PP1M and PP3M, recurrence was observed on average 58 days after oral paliperidone discontinuation, 172 days after PP1M discontinuation, and 395 days after PP3M discontinuation (Weiden et al., 2017). In this study and its predecessor DB study (Najarian et al., 2022), the incidence of relapse was rather low, hence the median time to relapse was not estimable.

As this was a single-arm study, the absence of a control arm/reference group was a potential limitation. Data were collected in local laboratories and electrocardiogram was not standardized, which would be characteristic of real-world conditions rather than as part of the typical phase 3 clinical study. Moreover, in this study, only relapse-free participants with schizophrenia who had completed the previous DB study were included, which may not fully represent the target population, thus limiting the generalizability of results. Finally, since the extension study was limited to 6 participating countries from a more expansive randomized DB study, data may have been affected by confounding demographic factors.

The twice-a-year administration of PP6M in this OLE showed continued efficacy with no new safety concerns over 2 years. The study had a high completion rate, and the results were consistent with the known profile of paliperidone palmitate products.

## CONCLUSIONS

The results of this OLE study support the long-term safety and efficacy of PP6M administration up to 24 months under circumstances similar to real-world conditions in participants with schizophrenia. Clinical improvements demonstrated in the DB study were maintained in this OLE study with stable scores of PANSS total, CGI-S, and PSP scales and very few relapses. Despite the larger volume of PP6M doses administered in the gluteal muscle compared with PP1M or PP3M, no new safety concerns were identified over the 2-year OLE period.

## Supplementary Material

pyad028_suppl_Supplementary_Material

## Data Availability

The data sharing policy of Janssen Pharmaceutical Companies of Johnson & Johnson is available at https://www.janssen.com/clinical-trials/transparency. As noted on this site, requests for access to the study data can be submitted through Yale Open Data Access [YODA] Project site at http://yoda.yale.edu. D. Najarian et al. Abstract submitted to European Psychiatry Association (EPA) 2023.

## Interest Statement

D.N. was an employee of Janssen Scientific Affairs, LLC and owned stock/stock options in Johnson & Johnson. I.T. and R.K.K. are employees of Janssen Research & Development, LLC, and own stocks/stock options in Johnson & Johnson. U.R. is an employee of Janssen Research and Development, Switzerland and owns stock/stock options in Johnson & Johnson. S.A. is affiliated with Dural Consulting, LLC and was a consultant for Janssen when this work was performed. S.G. is a consultant and advisor for Janssen, Gedeon Richter-Recordati, and Angelini and also reports fees as speaker in congresses and webinars for Angelini, Gedeon Richter-Recordati, Janssen, Lundbeck, Sunovion, Recordati, Rovi, and Boehringer Ingelheim. H.F.L. received research grants and principal investigator fee from Novartis, Eli Lilly, Lundbeck, Servier, AstraZeneca, Wyeth, Pfizer, Otsuka, Takeda, Sunovion, Roche, and Janssen Pharmaceutical and reports fees as a speaker from Servier, Abbot, Raymonds, Raffo, Temis Lostalo, and Janssen Pharmaceutical. PZ has nothing to disclose.
