# Supplementary material for: Long-Term Efficacy and Safety of Paliperidone 6-Month Formulation: An Open-Label 2-Year Extension of a 1-Year Double-Blind Study in Adult Participants With Schizophrenia
Source: Int J Neuropsychopharmacol. 2023 Jul 22;26(8):537–44. doi: 10.1093/ijnp/pyad028 (PMC10464922; doi:10.1093/ijnp/pyad028)
Supplement: pyad028_suppl_Supplementary_Material [file pyad028_suppl_supplementary_material.docx]

**SUPPLEMENTARY MATERIAL**

**Table S1. Summary of Efficacy Endpoints Among Subgroups PP3M/PP6M and PP6M/PP6M: Change From Baseline to Endpoint of Open-label Extension**

| Efficacy endpoints, mean (SD) | Baseline | Endpoint | Change from baseline |
| --- | --- | --- | --- |
| CGI-S scale | | | |
| PP3M/PP6M | 2.9 (0.83) | 2.7 (0.96) | –0.1 (0.44) |
| PP6M/PP6M | 2.8 (0.78) | 2.8 (0.91) | 0.0 (0.53) |
| PSP scale | | | |
| PP3M/PP6M | 71.5 (11.84) | 72.9 (12.84) | 1.2 (7.89) |
| PP6M/PP6M | 71.5 (10.79) | 71.8 (12.75) | 0.2 (7.28) |
| PANSS total score | | | |
| PP3M/PP6M | 50.2 (10.77) | 48.6 (11.53) | –1.4 (6.09) |
| PP6M/PP6M | 49.4 (10.40) | 50.6 (13.43) | 1.6 (8.93) |

Abbreviations: CGI-S, Clinical Global Impression-Severity; PANSS, Positive and Negative Symptom Scale; PSP, Personal and Social Performance; PP3M, paliperidone palmitate 3-month product; PP6M, paliperidone palmitate 6-month product; SD, standard deviation.

**Table S2. Safety: Injection Site Evaluation Over Time**

|  | Baseline  (n=178) | Endpoint  (n=175) |
| --- | --- | --- |
| Erythema/Redness, n (%) | | |
| Absent | 178 (100.0) | 175 (100.0) |
| Mild | 0 | 0 |
| Moderate | 0 | 0 |
| Severe | 0 | 0 |
| Induration/Swelling, n (%) | | |
| Absent | 176 (98.9) | 172 (98.3) |
| Mild | 2 (1.1) | 2 (1.1) |
| Moderate | 0 | 0 |
| Severe | 0 | 1 (0.6) |
| Tenderness, n (%) | | |
| Absent | 175 (98.3) | 168 (96.0) |
| Mild | 3 (1.7) | 6 (3.4) |
| Moderate | 0 | 0 |
| Severe | 0 | 1 (0.6) |

**Table S3. Summary of Efficacy Results: Change From Baseline to Endpoint of Open-label Extension vs. Baseline Double-blind Study to End of Open-label Extension**

| Efficacy endpoints, mean (SD) | Change from baseline to endpoint of OLE | Change from baseline (DB) to endpoint of OLE |
| --- | --- | --- |
| CGI-S scale | 0.0 (0.51) | –0.2 (0.54) |
| PSP scale | 0.5 (7.47) | 3.0 (8.60) |
| PANSS total score | 0.7 (8.22) | –3.2 (9.38) |

Abbreviations: CGI-S, Clinical Global Impression-Severity; DB, double-blind; OLE, open-label extension; PANSS, Positive and Negative Symptom Scale; PSP, Personal and Social Performance; PP3M, paliperidone palmitate 3-month product; PP6M, paliperidone palmitate 6-month product; SD, standard deviation.

**Table S4. Permitted Oral Antipsychotic Medications and Corresponding Doses**

|  | Oral Risperidone | Oral Paliperidone ER |
| --- | --- | --- |
| Moderate dose (700 mg eq. PP6M) | 1 – 2 mg/day | 1.5 – 3 mg/day |
| High dose (1000 mg eq. PP6M) | 1 – 3 mg/day | 1.5 – 6 mg/day |

Note: Maximum duration of co-administration with PP6M was 2 weeks. Within a single 6-month injection cycle, if the participant did not meet relapse criteria, an additional 2 weeks of oral antipsychotic was administered continuously for a maximum of 4 weeks. If, in the investigator’s judgment, there was a clinical need for oral antipsychotic supplementation for more than 4 continuous weeks, the study intervention was discontinued, and the participant was withdrawn. Use of oral antipsychotic medications other than those listed above was prohibited.

Abbreviations: ER: Extended release; PP6M, paliperidone palmitate 6-month product

**Figure S1. Median Prolactin Values Over Time by Gender**


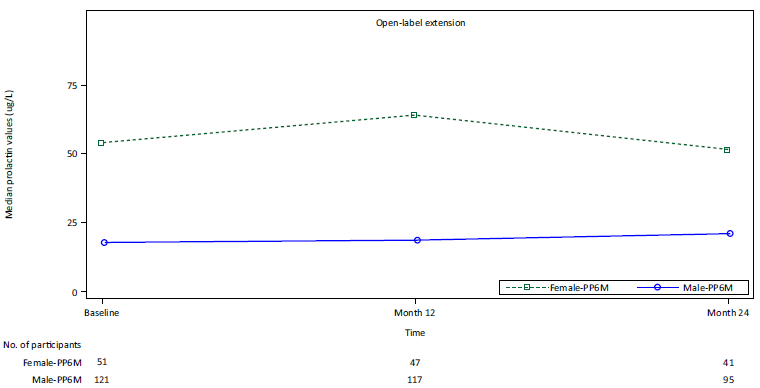


Abbreviation: PP6M, PP6M, paliperidone palmitate 6-month product.
